# Global market trends of flavour capsule and menthol cigarettes in 78 countries, 2010-2020

**DOI:** 10.1093/eurpub/ckac129.037

**Published:** 2022-10-25

**Authors:** CN Kyriakos, D Qi, K Chang, AA Laverty, FT Filippidis

**Affiliations:** Department of Primary Care and Public Health, Imperial College London, London, UK

## Abstract

**Background:**

Flavoured tobacco products, including innovative flavour capsule cigarettes (FCCs) and menthol cigarettes, can increase appeal and encourage smoking initiation and use. Global epidemiological data on these products are scarce.

**Methods:**

This study examined market trends of FCCs and menthol (non-capsule) cigarettes across 78 countries from 2010 to 2020 and assessed ecological-level factors associated with market shares of these products. Market share and retail volume data came from Euromonitor Passport and country-specific sociodemographic data come from the WHO and World Bank. Adjusted linear fixed effects panel regression analyses were used to evaluate the relationship between predictors variables and market outcomes.

**Results:**

Global total retail volume of FCCs increased over time (0.2% in 2010 to 4.5% in 2020) and market share was associated with year and unemployment rate and inversely associated with % urban population and smoking prevalence (p < 0.001). In contrast, menthol retail volume decreased over time (5.0% to 3.8%) and market share was associated with % urban population (p = 0.001) and inversely associated with year (p = 0.004) and unemployment rate (p = 0.017). The greatest market increase of FCCs was observed in the Americas region and among upper-middle income countries. In Europe, there was a decrease of 0.64 percentage points from 2019 to 2020.

**Conclusions:**

Overall, FCCs experienced substantial global growth in the recent decade, with the exception of Europe whose slight decrease may be attributable to the European Union ban on flavours in cigarettes. Findings indicate that there is a need for increased efforts to address flavours and innovative features used in tobacco products, which are known to appeal to youth.

**Key messages:**

• This study contributes to global monitoring of tobacco products.

• Findings can be used by advocates and policy makers to support countries in adopting measures to ban flavoured tobacco products.

